# Gut Microbiota and Colorectal Cancer Development: A Closer Look to the Adenoma-Carcinoma Sequence

**DOI:** 10.3390/biomedicines8110489

**Published:** 2020-11-10

**Authors:** Marco Vacante, Roberto Ciuni, Francesco Basile, Antonio Biondi

**Affiliations:** Department of General Surgery and Medical-Surgical Specialties, University of Catania, Via S. Sofia 78, 95123 Catania, Italy; ciuni.r@gmail.com (R.C.); fbasile@unict.it (F.B.); abiondi@unict.it (A.B.)

**Keywords:** colorectal cancer, gut microbiota, colorectal adenoma, polyps, bacteria

## Abstract

There is wide evidence that CRC could be prevented by regular physical activity, keeping a healthy body weight, and following a healthy and balanced diet. Many sporadic CRCs develop via the traditional adenoma-carcinoma pathway, starting as premalignant lesions represented by conventional, tubular or tubulovillous adenomas. The gut bacteria play a crucial role in regulating the host metabolism and also contribute to preserve intestinal barrier function and an effective immune response against pathogen colonization. The microbiota composition is different among people, and is conditioned by many environmental factors, such as diet, chemical exposure, and the use of antibiotic or other medication. The gut microbiota could be directly involved in the development of colorectal adenomas and the subsequent progression to CRC. Specific gut bacteria, such as *Fusobacterium nucleatum*, *Escherichia coli,* and enterotoxigenic *Bacteroides fragilis,* could be involved in colorectal carcinogenesis. Potential mechanisms of CRC progression may include DNA damage, promotion of chronic inflammation, and release of bioactive carcinogenic metabolites. The aim of this review was to summarize the current knowledge on the role of the gut microbiota in the development of CRC, and discuss major mechanisms of microbiota-related progression of the adenoma-carcinoma sequence.

## 1. Introduction

Colorectal cancer (CRC) is a leading cause of cancer mortality worldwide with approximately 900,000 deaths every year, and the increasing age-standardized incidence rate of CRC in most countries represents an important public health challenge [1]. Indeed, the global incidence of CRC was 1.8 million (95% UI 1.8–1.9) in 2017, with an age-standardized incidence rate of 23.2 per 100,000 person-years that raised by 9.5% (4.5–13.5) between 1990 and 2017 [2]. There is wide evidence that CRC risk is highly modifiable through diet and lifestyle [3]. Several studies suggested that a significant number of CRC cases could be prevented by regular physical activity, keeping a healthy body weight, and following a healthy and balanced diet [4,5,6].

Around 60–90% of sporadic CRCs arise via the traditional adenoma-carcinoma pathway, starting as premalignant lesions represented by conventional, tubular, or tubulovillous adenomas [7]. Cancers that derive from this pathway are frequently associated with male sex, and located in the distal colon. These tumors are characterized by chromosomal instability (CIN), inactivating mutations or losses in the adenomatous polyposis coli (APC) tumor suppressor gene, and in some cases mutations in the *KRAS* oncogene, *SMAD4*, *PIK3CA*, and *TP53* genes [8,9].

The term “gut microbiota” indicates the collection of microorganisms (bacteria, archaea and eukarya) colonizing the human gastrointestinal tract. Overall, the number of these microorganisms has been calculated to exceed 10^14^, with a ratio of human:bacterial cells closer to 1:1 [10,11]. The gut bacteria play a crucial role in regulating host metabolism (i.e., absorption of indigestible carbohydrates and fat-soluble vitamins, and stimulation of innate and cell-mediated immunity) and also contribute to preserve intestinal barrier function and an effective immune response against pathogen colonization [12,13,14]. The microbiota composition is different among people, and is conditioned by many environmental factors, such as diet, chemical exposure, and the use of antibiotic or other medication [15].

Several studies suggested that the gut microbiota could be directly involved in the development of colorectal adenomas and the subsequent progression to CRC [16]. Patients with CRC could present changes in microbial composition and ecology, and functional studies in animal models underlined the importance of certain bacteria, such as *Fusobacterium nucleatum*, *Escherichia coli,* and *Bacteroides fragilis,* in colorectal carcinogenesis [17,18]. Possible mechanisms of CRC progression may include DNA damage, promotion of chronic inflammation, and release of bioactive carcinogenic metabolites [19,20,21].

The aim of this review was to summarize the current knowledge on the role of the gut microbiota in the development of CRC, including major mechanisms of microbiota-related progression of the adenoma-carcinoma sequence.

## 2. Risk Factors for the Development of Adenomas and CRC

Genetic alterations play a key role in the progression of adenomas to CRC; for instance, mutations may occur in oncogenes (i.e., *KRAS*), tumor suppressor genes such as *APC*, *p53*, and *CTNNB1*, as well as in pathways associated with CpG island methylation (CIMP), mismatch repair (MMR), and chromosomal and microsatellite instability (CIN and MSI) [22,23,24]. Ageing and family history have been also correlated with higher risk of adenomas and CRC [25,26,27].

It has been suggested that genetic predisposition and somatic mutations in combination with environmental factors could be responsible for CRC, in the way of a complex disease [28,29,30]. Lifestyle and dietary habits represent the most common environmental factors associated with colorectal adenomas and CRC [31,32,33]. Even if it is difficult to analyze the single dietary risk factors in epidemiological studies, preclinical animal models have shown the key role of nutrition in tumor development [34,35]. Nutrition may affect the incidence, natural progression and therapeutic response of cancer, modulating the release of endocrine factors, modifying inflammatory and immunological pathways, or by changing the gut microbiota composition [36,37,38].

An increased risk of adenomas and CRC has been observed in subjects consuming diets high in red meat or processed meat, food with a high glycemic index, salt and alcohol, and low daily water and fiber intake [39,40]. On the contrary, the consumption of white meat, vegetables and fish oils with a high omega-3 polyunsaturated fatty acids (PUFA) to omega-6 PUFA ratio could lower the risk of CRC [41,42,43]. A diet rich in fiber, vitamin B6, C, D, E, folic acid, magnesium and selenium, has also been suggested to decrease the risk of CRC [44]. Other risk factors that may contribute to the development of CRC are obesity, smoking, male sex, non-hispanic black ethnicity, and lack of physical activity [45,46,47].

There is growing evidence that diet may select for the microbiota composition, thus regulating many beneficial or harmful effects of gut bacteria [15,48]. For instance, dietary fiber are able to stimulate the colonic microbial production of anti-proliferative and counter carcinogenic substances, especially butyrate [49]. The adoption of a healthy lifestyle, and a diet rich in fiber, vegetables and fruit, could decrease the risk of CRC. Moreover, a recent study showed that higher fiber intake after the diagnosis of non-metastatic CRC (non-mCRC) was associated with decreased CRC-specific and overall mortality. Indeed, an increased fiber intake after CRC diagnosis could give supplementary advantages to patients with CRC due to the interaction with gut microbiota [50,51].

## 3. Dysbiosis, Inflammation and Toxic Bacterial Metabolites

The adenomas are the most frequent premalignant precursor lesions of almost all the sporadic CRCs [52]. Up to 40% of individuals aged 60 years or older may present adenomatous polyps, with a transformation rate into CRC of approximately 0.25% per year [53,54]. Inactivating mutations of the *APC* gene are considered as the initial step of the adenoma-carcinoma sequence. A loss of *APC* gene activity results in the accumulation of β-catenin, that leads to abnormal cell proliferation, and formation of adenomatous polyposis [55]. There is evidence that an interaction between gut microbiota and genetic could contribute to the genetic pattern of the adenoma-carcinoma sequence; indeed, bacterial drivers could be responsible for the initiation of precancerous lesions and the subsequent accumulation of gene mutations [56,57,58].

Chronic inflammation has also been suggested to play a crucial role in many aspects of CRC initiation, promotion, and progression [59,60]. A meta-analysis confirmed the association between circulating levels of C-reactive protein (CRP), a non-specific marker of systemic inflammation, and risk of colorectal adenoma [61]. Also, higher levels of pro-inflammatory cytokines, such as tumor necrosis factor-alpha (TNF-α) and interleukin-6 (IL-6), have been observed within adenoma tissues as an expression of an inflammatory state. TNF-α and IL-6 are also involved in cell growth, differentiation, and apoptosis [62,63].

At the phyla level, the colonic microbiota of healthy individuals usually shows a predominance of Gram-positive Firmicutes and Gram-negative Bacteroidetes, with a less presence of Verrucomicrobia and Actinobacteria. The Firmicutes phylum is represented by more than 200 different genera including *Clostridium*, *Lactobacillus*, *Enterococcus, Bacillus*, and *Ruminicoccus*. The Actinobacteria phylum mainly consists of the *Bifidobacterium* genus [64,65]. Variation in the composition of gut microbiota between phenotypically similar and healthy subjects may be influenced by age, gender, genetics, diet and diseases [66].

Some studied reported abnormalities in the normal bacterial community composition, known as dysbiosis, in CRC patients [67]. Dysbiosis of the gut microbiota is characterized by the reduction in commensal bacterial species (i.e., butyrate-producing bacteria) and the growth of detrimental bacterial strains (i.e., pro-inflammatory opportunistic pathogens) [68].

**Figure 1:**
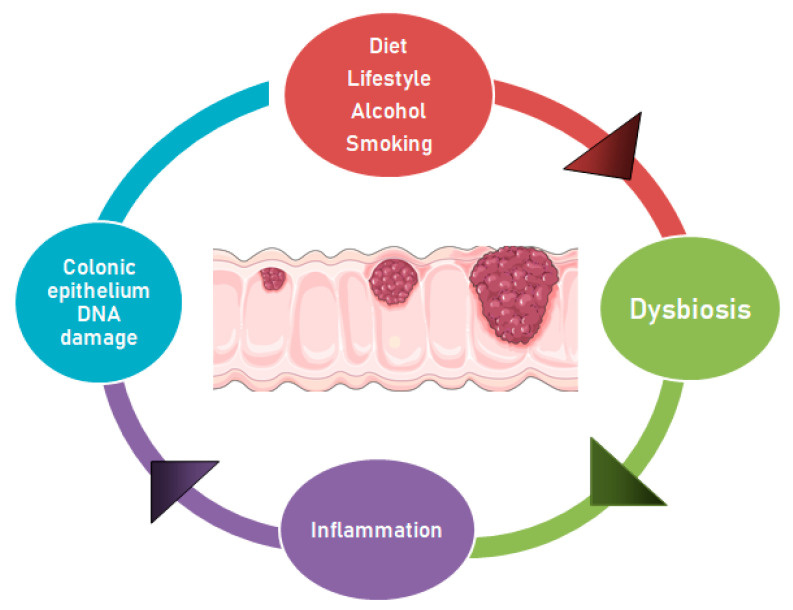
Dysbiosis and other factors contributing to the adenoma-carcinoma progression. The adenoma-carcinoma progression may occur because of the genomic instability caused by alterations in the gut microbiota. These changes may be supported by diet and lifestyle, which promote dysbiosis, inflammatory state and epithelial DNA damage, thus contributing to CRC development. The carcinogenesis leads to gut niche changes, which may favor the proliferation of opportunistic pathogens.

Changes in the balance of commensal bacteria may lead to a raise in mucosal permeability, bacterial translocation, and activation of factors of the innate and adaptive immune system to stimulate chronic inflammation [69]. Over-expression of proinflammatory cytokines, such as IL-12, IL-23, IFNγ and TNF-α by dendritic cells, macrophages, and natural killer (NK) cells, may further promote the activation of T and B cells and different inflammatory mediators. The activation of signaling pathways by transcription factors such as NF-κB and signal transducer and activator of transcription 3 (STAT3) in colonic epithelial cells, the production of reactive oxygen species (ROS) and the related oxidative stress, DNA damage, and abnormal cell proliferation, may favor the development of colorectal adenomas and cancer [70,71,72] (Figure 1).

During chronic inflammation, there is a general imbalance in the gut due to release of toxic compounds and procarcinogens. Actually, an abnormal generation of bacterial metabolites directly involved in tumor metabolism, such as polyamines and short-chain fatty acids (i.e., butyrate, propionate and acetate), has been observed in patients with adenomas and CRC [15,73]. Under homeostasis, the gut microbiota is metabolized to generate many beneficial compounds for the host, whereas under an unbalanced state, the bacterial growth and health of the host may be negatively influenced [74].

The microbiota initiates and supports the hypoxic environment of the gut that is fundamental for nutrient absorption, epithelial barrier function, and immune response. The response to hypoxia is regulated by hypoxia-inducible factors (HIFs), which modulate the expression of genes, including the ones involved in metabolism, that promote adaptation to hypoxia. Chronic HIF activation may aggravate disease conditions, leading to intestinal damage, inflammation, and CRC [75,76,77].

Overall, the fermentation of carbohydrates produces short-chain fatty acids, especially butyrate, which can be utilized by the host and shows antineoplastic properties, while proteolytic fermentation generates ammonia, sulphides, phenols, and cresols, which may exert a pro-inflammatory effect, increase tissue permeability and in turn contribute to the development of adenomas and CRC [78,79]. Great amounts of specific strains of bacteria may lead to the generation of other substances with anti- and/or pro-carcinogenic effects, such as enterotoxins, B vitamins, urolithins, cyclomodulins, lignans, and equol [16,80].

Changes of the microbiota profile in adenomas could enhance the production of primary and secondary bile acids, as well as sucrose, lipid, starch, and phenylpropanoid metabolism, thus supporting an intestinal environment that favors the growth of bile-resistant and sulfidogenic microorganisms including *Desulfovibrio* and *Bilophilia* [81,82].

It is well recognized that hydrogen sulfide (H_2_S) generated by bacteria in the gut is related to adenoma development and eventually CRC [83]. Many anaerobic bacterial strains such as *Salmonella enterica, Clostridia, Escherichia coli*, and *Enterobacter aerogenes* are able to convert cysteine to H_2_S, ammonia and pyruvate by cysteine desulfhydrase; moreover some gut bacteria (i.e., *Escherichia coli, Salmonella, Enterobacter, Staphylococcus, Bacillus, Klebsiella, Corynebacterium,* and *Rhodococcus*) may generate H_2_S by sulfite reduction [84]. H_2_S modulates inflammation, ischemia and/or perfusion injury and motility, and exerts a toxic activity on the colonic epithelium [85]. Phenolic substances such as amines, N-nitroso compounds (NOCs) found in processed meat, may also exert toxic activities favoring carcinogenesis [86,87].

Colibactin is a genotoxin produced by certain strains of bacteria, such as B2 phylogroup *E. coli* strains that colonize the human gut [88]. The synthesis of colibactin by the polyketide synthetase (*pks*) genomic island, especially in members of the family *Enterobacteriaceae,* may lead to chromosomal instability and DNA damage in eukaryotic cells, apoptosis of immune cells, and in turn the development of CRC [89].

## 4. Specific Bacteria Associated with Colorectal Adenoma and Cancer Development

Numerous studies have identified tumour-specific bacteria present in colorectal mucosal and/or faecal samples, and not detectable in healthy controls or tumour tissue versus the bordering healthy mucosa [90] (Table 1). A metagenome-wide association study (MGWAS) on stools from advanced adenoma and CRC patients and from healthy individuals, detected microbial genes, strains and functions enriched in each group. High consumption of red meat relative to fruits and vegetables seems to be associated with development of specific bacteria that could contribute to a more hostile intestinal milieu [91]. In general, microbial species associated with CRC development are represented by specific strains of *Escherichia coli*, *Streptococcus gallolyticus*, *Bacteroides fragilis*, *Fusobacterium nucleatum*, and *Enterococcus faecalis* among others [16].

Hale et al. observed significant abundances of multiple taxa in subjects with adenomas, such as *Bilophila, Desulfovibrio,* pro-inflammatory bacteria in the genus *Mogibacterium*, and *Bacteroidetes* spp. On the other hand, *Veillonella*, Firmicutes (class Clostridia), and Actinobacteria (family *Bifidobacteriales*) were more represented in patients without adenomas [81].

A study by Peters et al. analyzed for the first time the link between the gut microbiota and specific colorectal polyp types in 540 subjects, and showed that conventional adenomas (CA) cases had lower species diversity in faeces compared to controls (*p* = 0.03), especially with regard to advanced CA cases (*p* = 0.004). Only subjects with distal or advanced CA showed significant differences in general microbiota composition compared to controls (*p* = 0.02 and *p* = 0.002). Faeces of CA cases were characterized by the reduction in *Clostridia* from families *Ruminococcaceae*, *Clostridiaceae*, and *Lachnospiraceae*, and the increase in the classes Gammaproteobacteria and Bacilli, order Enterobacteriales, and genera *Streptococcus* and *Actinomyces*. There were not significant differences between sessile serrated adenoma (SSA) and hyperplastic polyps (HP) cases in diversity or composition compared to controls [92].

Feng et al. detected a great amount of *Bacteroides* and *Parabacteroides*, together with *Bilophila wadsworthia*, *Lachnospiraceae bacterium*, *Alistipes putredinis*, and *Escherichia coli* in CRC compared with both healthy and advanced adenoma. Also, gut commensals such as *Bifidobactium animalis* and *Streptococcus thermophilus*, were diminished in stools from adenoma or CRC patients, thus highlighting a divergence from healthy microbiota. Patients with advanced adenoma or CRC seem to be lacking in lactic acid-producing commensals such as *Bifidobacterium* that could facilitate epithelium regeneration and inhibition of opportunistic pathogens [91].

### 4.1. Fusobacterium nucleatum

*F. nucleatum* is an oral symbiont, and opportunistic pathogen that has been detected in intestinal cancers [93,94]. *F. nucleatum* may enhance CRC carcinogensis by stimulating the production of interleukin (IL)-17F/21/22/23/31/cluster of differentiation (CD)40L and protein expression of phospho-STAT3 (p-STAT3), p-STAT5, and phospho-extracellular regulated protein kinases (p-ERK)1/2 [95]. A great amount of *Fusobacteria* has been observed in SSA [108,109]; a study by Yu et al. reported that the prevalence of invasive *Fusobacteria* within proximal SSAs (78.8%) and HPs (65.7%) was significantly more elevated than that of proximal and distal traditional adenomas (28.9% and 24.4% respectively; *p* < 0.05) [96]. The presence of *F. nucleatum* has been associated with poor prognosis in CRC patients and development of chemoresistance [97,98]. *F. nucleatum* binds E-cadherin in the clonic epithelium and stimulates colorectal carcinogenesis through the fusobacterial adhesin FadA [110,111]. The interplay between Gal-GalNAc, a host polysaccharide, with fusobacterial lectin (Fap2) may promote the increase of *F. nucleatum* in colorectal adenoma and cancer [112]. A study by Mima et al. showed that multivariable hazard ratios (HRs) for CRC-specific mortality in *F. nucleatum*-low subjects and *F. nucleatum*-high subjects, compared with *F. nucleatum*-negative subjects, were 1.25 (95% C.I. 0.82 to 1.92) and 1.58 (95% C.I. 1.04 to 2.39), respectively (*p* for trend = 0.020). The quantity of *F. nucleatum* was correlated with microsatellite instability (MSI)-high (multivariable odd ratio (OR), 5.22; 95% CI 2.86 to 9.55) independent of the presence of CIMP and BRAF mutation. A significant association between CIMP and BRAF mutation with *F. nucleatum* was observed only in univariate analyses (*p* < 0.001) but not in multivariate analysis that adjusted for MSI status [97].

Yang et al. observed that an infection of CRC cells lines (HCT116, HT29, LoVo, and SW480) with *F. nucleatum* increased cell growth, invasiveness, and capability to form xenograft cancers in mice. *F. nucleatum* promoted Toll-like receptor 4 (TLR4) signaling to myeloid differentiation factor 88 (MYD88), activating NFκB signaling pathways and increasing the expression of microRNA-21 (miR21), which reduced the levels of the RAS GTPase p21 protein activator 1 (RASA1). Shorter survival times were observed for tumors with high amounts of *F. nucleatum* DNA and miR21 [113].

It has been also observed that *F. nucleatum* may promote LC3-II protein expression, autophagy pathway, and autophagosome production in CRC cells. *F. nucleatum* may favor the release of the autophagy-related proteins, pULK1, ULK1, and ATG7, contributing to the resistance to oxaliplatin and 5-fluorouracil regimens in CRC cells [98].

A study by Bullman et al. showed the persistance of F. nucleatum also in distal metastatic lesions of CRC patients. Administration of metronidazole in mice bearing a colon cancer xenograft decreased *F. nucleatum* load, tumor cell proliferation, and overall cancer development, thus suggesting that specific antibiotics could potentially be used to treat patients with *Fusobacterium*-associated CRC [114].

### 4.2. Streptococcus gallolyticus (Formerly S. bovis)

*Streptococcus gallolyticus subsp. gallolyticus* (SGG), formerly known as *S.* *bovis* biotype I, represents a common causative agent for bacteremia and endocarditis in older adults. Gut colonization by SGG is strongly correlated with the development of CRC [99,115]. Indeed, both American and European guidelines recommended colonoscopy in patients with SGG bacteremia [116,117].

A case-control study by Corredoira-Sánchez et al. carried out on 109 cases showed that the prevalence of CRC was higher in patients with SGG bacteremia compared to controls (70% vs. 32%; OR, 5.1; 95% CI 3.0–8.6). The study did not show significant differences when comparing nonadvanced adenomas (19% vs. 12%). However, significant differences were observed in advanced adenomas (40% vs. 16%; OR 3.5, 95% C.I. 2.0–6.1) and invasive CRC (12% vs. 5%, OR 2.9, 95% C.I. 1.2–6.9) [100].

A large epidemiological study by Butt et al. showed for the first time a statistically significant association between exposure to SGG antigens and CRC, and pointed out that the risk for CRC was stronger among subjects younger than 65 years [101].

Aymeric et al. observed that CRC-specific conditions may favor SGG colonization of the gut at the expense of commensal enterococci. Indeed, gut colonization by SGG is promoted by a bacteriocin called “gallocin”, which is enhanced by bile acids and may exert toxic activity to enterococci. Also, the stimulation of the Wnt pathway, and the reduced expression of the bile acid apical transporter gene *Slc10A2*, may act on the *APC* founding mutation, supporting the gut colonization by SGG [115].

### 4.3. Enterotoxigenic Bacteroides fragilis (ETBF)

*Enterotoxigenic B. fragilis* (ETBF) may support colorectal carcinogenesis by the production of pro-inflammatory cytokines and the stimulation of Wnt signaling. Expression of *B. fragilis* toxin (BFT), a 20 kDa metalloprotease produced by ETBF, is able to promote persistent colitis in mice, damage E-cadherin junctions, as well as stimulate B-catenin signaling and IL-8 production in colonic epithelial cells [118].

A study by Purcell et al. underlined the key role of ETBF in the development of colorectal low-grade dysplasia, tubular adenomas, and serrated polyps (*p*-values of 0.007, 0.027 and 0.007, respectively) [102]. Similar findings were reported in a study of patients with colonic adenomas that presented higher expression of the *B. fragilis* toxin gene (*bft*) associated with adenoma tissue compared to normal healthy mucosa [103].

Zamani et al. reported an increased positivity of ETBF in patients with precancerous and cancerous lesions compared to healthy controls. Higher ORs of ETBF were significantly associated with serrated lesions and adenoma with low-grade dysplasia. The most common subtype of *bft* gene was the *bft1* gene, followed by the *bft2* gene. An assessment of ETBF could represent a marker of CRC prognosis, especially in the precancerous lesions, and could be used for the screening of these conditions [104].

### 4.4. Enterococcus faecalis

*E. faecalis* is a Gram-positive commensal bacterium, that may be responsible for human disease through translocation from intestinal wall, oral cavity, and genito-urinary mucosa, leading to a systemic infection [119]. *E. faecalis* represents one of the most frequent causes of infection in older adults, and some studies underlined its importance for the development of cancer [120]. It has also been reported an association between enterococcal endocarditis and hidden CRC [119,121]. On the other hand, *E. faecalis* showed anti-inflammatory properties and probiotic activity, and is frequently administered in subjects with chronic sinusitis and bronchitis or in infant acute diarrhea [122].

Actually, there is no consensus on the role of *E. faecalis* in CRC: some studies highlighted its protective role or no role in CRC, whereas others reported potential pro-carcinogenic effects [123].

A study by Viljoen et al. carried out on 55 patients, did not highlight any significant clinical association between *E. faecalis* and CRC. However, the same study showed a relevant association bewteen clinicopathological features of CRC and *Fusobacterium* spp. and ETBF [105]. Miyamoto et al. observed that heat-killed *E. faecalis* strain EC-12 could suppress intestinal polyp development in Apc mutant Min mice. Administration of heat-killed EC-12 reduced the levels of c-Myc and cyclin D1 mRNA expression in intestinal polyps, by blocking the transcriptional activity of the T-cell factor/lymphoid enhancer factor [124].

*E. faecalis* could play a role in inducing CRC by activation of Wnt/β-catenin signaling and induction of pluripotent transcription factors linked to dedifferentiation. Indeed, exposure of murine primary colon epithelial cells to *E. faecalis*-infected macrophages contributed to CRC initiation through gene mutation, chromosomal instability, and endogenous cell transformation, which involved the transcription factors c-Myc, Klf4, Oct4, and Sox2i [125].

Perhaps, these controversial data could be explained taking into account the different geographical origin of the isolated strain, and dysbiosis due to the use of antibiotics or changes in diet [126,127].

### 4.5. Escherichia coli

Classification of the Gram negative bacterium *E. coli* includes 8 phylogenetic groups (A, B1, B2, C, D, E, F and clade I). Commensal strains are commonly represented by A and B1 groups, being the largest part of the fecal flora of healthy individuals. Extraintestinal pathogenic strains (ExPEC) include mainly B2 and D groups, and may be responsible for many extraintestinal infections, due to the achievement of numerous virulence factors that potentially support the colonization of extraintestinal tissues [128]. However, both commensals and ExPEC are considered as a part of the normal gut microbiota in healthy subjects [129].

There is evidence that *E. coli* could play a role in the development of CRC [106,130]. Indeed, some patients with CRC may show an excessive growth of *E. coli* strains, mainly B2, characterized by high expression of virulence genes, including those encoding toxins and effectors that may induce carcinogenesis, such as colibactin, cytolethal distending toxins, cytotoxic necrotizing factors, and cycle-inhibiting factor [131,132]. In vitro studies showed that colibactin could be involved in DNA alkylation on adenine residues, leading to double-strand breaks [133,134]. Pleguezuelos-Manzano et al. demonstrated that exposure to genotoxic *pks* + *E. coli*, could be responsible for specific mutational signature in human intestinal organoids; indeed, an identical mutational signature was observed in 5876 human cancer genomes from two independent study cohorts, mostly in CRC [135].

Ambrosi et al. analyzed 272 *E. coli* isolates from colonoscopy biopsies, and showed that *E. coli* strains colonizing adenomatous polyps were characterized by specific phenotypes compared to those from normal mucosa, which included lack of motility, moderate to strong biofilm forming activity, and poor proteolytic capability [106].

In a study by Iyadorai et al. *pks* + *E. coli* was detected more frequently in CRC patients compared to healthy subjects. In vitro assays carried out on primary colon epithelial (PCE) and CRC (HCT116) cell lines, highlighted that the cytopathic effect of *pks* + *E. coli* strains could support the initiation and development of CRC [107].

## 5. Future Perspectives

Modulation of the gut microbiota, aiming to reverse microbial dysbiosis, could represent a new tool for prevention and treatment of CRC. The strategies could include the use of probiotics, prebiotics, postbiotics, antibiotics, and fecal microbiota transplantation (FMT) [136,137,138,139].

Overall, the effects of microbiota modulation on CRC prevention could be due to many mechanisms, such as the suppression of inflammatory state, stimulation of apoptosis of early cancer cells, re-establishment of intestinal barrier function and correction of microbiota composition [140,141]. Also, manipulation of the gut microbiota could alleviate chemotherapy-induced side effects, such as mucositis, as confirmed by a decreased incidence of diarrhea and weight loss after the administration of several probiotics strains in animal models [142,143].

There is growing evidence that modifications of microbial abundances in some pathological conditions could affect their co-abundance interactions; indeed, Chen et al. observed specific gut microbial co-abundance networks in patients with inflammatory bowel disease (IBD) and obesity. These findings underlined the importance of microbial dysbiosis in the pathogenesis of some diseases, and suggested that even the development of CRC could share similar mechanisms [144,145,146].

Promising preclinical studies suggested that modulation of gut microbiota could increase therapeutic efficacy of anticancer drugs. There is evidence that the administration of antibiotics could lead to clinical benefits to CRC patients by gut microbiota depletion and subsequent reduction of chemotherapeutic resistance. Indeed, a study by Geller et al. observed that intratumor bacteria could favor gemcitabine resistance through enzymatic inactivation, and therefore the administration of a gemcitabine-ciprofloxacin combination therapy could enhance the efficacy of chemotherapy [147].

Some studies demonstrated that the gut microbiota is also able to affect chemotherapy and/or immunotherapy efficacy by modulating immune response [148]. Oral administration of some probiotics, such as *Bifidobacterium* spp. and *Akkermansia muciniphila*, or FMT from treatment-responsive patients, stimulated the programmed cell death protein 1 ligand 1 (PD-L1)-based immunotherapy, thus blocking cancer development through the increase of dendritic cell and T cell response [149,150,151].

There is growing evidence that microbial shift markers could be used succesfully for non-invasive early diagnosis and/or prognostic assessment of CRC and advanced adenomas [81,152]. Mangifesta et al. performed a metataxonomic analysis based on 16S rRNA gene sequencing approach, and showed that some microbial taxa such as *Bacteroides*, *Faecalibacterium*, and *Romboutsia*, seem to be reduced in cancerogenic mucosa and in adenomatous polyps, thus representing potential new biomarkers of early carcinogenesis. Furthermore, the detection of high amounts of *F. nucleatum* in polyps, underlined the key role of this microorganism as a microbial biomarker for early diagnosis of CRC [153].

A study by Hale et al. showed that the composition of the gut microbiota in subjects with adenomas is significantly different from that of healthy subjects, and is similar to the microbiota of subjects with CRC. These changes could be a consequence of the Western diet and could result in metabolic changes leading to intestinal cellular damage and mutagenesis [81,154].

The combined assessment of heterogeneous CRC cohorts detected reproducible microbiota biomarkers and disease-predictive models that could represent useful tools for clinical prognostic tests and future research. A meta-analysis of 969 stool metagenomes carried out using data from five open access datasets and two new cohorts, showed that the gut microbiota in CRC was characterized by more richness than controls (*p* < 0.01), partly due to the growth of some species originating from the oral cavity. The results also highlighted an association between gluconeogenesis, putrefaction and fermentation processes with CRC, while the starch and stachyose degradation were associated with controls. A significant association between microbiota choline metabolism and CRC was also observed (*p* = 0.001) [155]. Another meta-analysis of eight stool metagenomic studies of CRC (n = 768) from different geographical areas, reported a significant enrichment in a group of 29 species in CRC metagenomes (FDR < 1 × 10^−5^). An elevated production of secondary bile acids from CRC metagenomes, higher expression of mucin and protein catabolism genes and reduction of carbohydrates degradation genes were observed, thus underlying a metabolic relationship between gut microbiota in CRC and a diet rich in meat and fat [156].

A study by Poore et al. carried out on The Cancer Genome Atlas (TCGA) detected specific microbial signatures in blood and tissue of different types of tumors, including CRC, which were predictive for patients with stage Ia-IIc tumor and tumors without any genomic modifications as detected by cell-free tumor DNA assessment. These findings could pave the way to a novel type of microbial-based CRC diagnostics [157].

Currently, there is a great limitation in availability of mouse models to study the interaction between gut microbiota and CRC. Zeb2^IEC-Tg/+^ (intestinal epithelial cell-specific transgenic expression of the epithelial-to-mesenchymal transition regulator Zeb2) mice represented the first and only microbiota-dependent CRC mouse model available so far. Specific characteristics of Zeb^2IEC-Tg/+^ mice included the presence of gut dysbiosis, and the preventive effect on carcinogenesis through the microbiota reduction by broad-spectrum antibiotics or germ-free rederivation [158].

## 6. Conclusions

In conclusion, detecting key relationships between diet, gut microbiota, and metabolites involved in the adenoma-carcinoma sequence could provide important basis for personalized medicine aimed at preventing and managing CRC. Secondary bile acids, H_2_S, and other bacterial metabolites could exert genotoxic activities and should be kept into account when investigating the adenoma and carcinoma development. Nonetheless, further studies are needed to evaluate the effects of diet, lifestyle, or medications on the gut metabolic environment and the microbiota. Finally, the identification of global microbiota signatures specific for CRC represents a promising tool in CRC diagnosis and therapy.

## Figures and Tables

**Table 1:** Studies of gut bacteria associated with the development of adenoma and/or CRC

Authors (Year).	Bacteria	Methods	Sample Size	Statistical Significance (*p* Value, Odds Ratio, and/or Hazard Ratio)	Clinical Evidence
Hale et al. (2017) [81]	*Bilophila*, *Desulfovibrio*, *Mogibacterium*, *Bacteroidetes* spp.	16S rRNA gene sequencing	233 adenomas, 547 controls	AUC of 0.6599, (*p* = 0.001)	Adenoma and CRC development
Kasai et al. (2016) [90]	*Actinomyces*, *Atopobium*, *Fusobacterium*, and *Haemophilus* spp.	T-RFLP and NGS	49 controls, 50 adenomas, 9 CRC (3/9 invasive cancer and 6/9 carcinoma in adenoma	*Actinomyces odontolyticus* (*p* = 0.007), *Bacteroides fragile* (*p* = 0.004), *Clostridium nexile* (*p* = 0.036), *Fusobacterium varium* (*p* = 0.022), *Haemophilus parainfluenzae* (*p* = 0.020), *Prevotella stercorea* (*p* = 0.022), *Streptococcus gordonii* (*p* = 0.014), and *Veillonella dispar* (*p* = 0.042)	Association with CRC development
Feng et al. (2015) [91]	*Bacteroides*, *Prevotella*, and *Parabacteroides* spp. *Alistipes putredinis*, *Bilophila wadsworthia*, *Lachnospiraceae bacterium*, *Fusobacterium*, *E. coli*	MGWAS on stools	55 controls, 42 advanced adenoma, 41 CRC	*p* = 0.005, *p* < 0.001 (among the groups respectively, Kruskal–Wallis test)	Development of advanced adenoma and CRC
Peters et al. (2016) [92]	Reduction in Clostridia (*Clostridiaceae*, and *Lachnospiraceae*), and enrichment in Bacilli and Gammaproteobacteria, (Enterobacteriales), *Actinomyces* and *Streptococcus*	16S rRNA gene sequencing	540 total: 144 CA, 73 serrated polyps, 323 polyp-free controls	CA *p* = 0.03; advanced CA *p* = 0.004. Distal or advanced CA vs. controls (*p* = 0.02 and *p* = 0.002)	Early stages of carcinogenesis and development of CAs
Li et al. (2016) [93]	*F. nucleatum*	FQ-PCR in CRC and normal tissues, FISH analysis (to confirm 22 cases)	101 CRC	CRC vs. controls: 0.242 (95% C.I. 0.178–0.276) vs. 0.050 (95% C.I. 0.023–0.067), *p* < 0.001	Association with CRC development and metastasis
Fukugaiti et al. (2015) [94]	*F. nucleatum* and *Clostridium difficile*	qRT-PCR	17 total: 7 CRC	*F. nucleatum* (*p* < 0.01); *Clostridium difficile* (*p* < 0.04)	Possible role of in CRC carcinogenesis
Yu et al. (2015) [95]	*Fusobacterium*, *Streptococcus* and *Enterococcus* spp.	Pyrosequencing of the 16S ribosome RNA (rRNA) from fecal samples	52 controls, 47 advanced adenoma, 42 CRC	Increase of the three bacteria groups during the adenoma-carcinoma sequence: *p* < 0.05. Increase of the Fusobacterial phylum: from normal (0.27%) to adenoma (0.61%) to CRC (1.69%) (*p* = 0.016)	*F. nucleatum* colonization in the gut may favor colorectal tumorigenesis
Yu et al. (2016) [96]	*F. nucleatum*	16S rRNA FISH	35 HPs, 33 SSAs, 48 proximal CRCs, and 10 matched metastatic lymph nodes	Higher *Fusobacterium* in proximal HPs and SSAs vs. proximal TAs and distal TAs (*p* < 0.05). Higher *Fusobacterium* in more proximal CRCs vs. distal CRCs (*p* < 0.05), and in matched metastatic lymph nodes vs. nonmetastatic lymph nodes (*p* < 0.001).	Carcinogenesis of proximal colon through the serrated neoplasia pathway. Less important role in the TA-carcinoma sequence.
Mima et al. (2016) [97]	*F. nucleatum*	Assessment of DNA in CRC tissue	1069 CRC in the Nurses’ Health Study and the Health Professionals Follow-up Study	HRs for CRC-specific mortality in *F. nucleatum*-low cases and *F. nucleatum*-high cases:1.25 (95% C.I. 0.82 to 1.92) and 1.58 (95% C.I. 1.04 to 2.39), respectively, (*p* for trend = 0.020). Association with MSI-high OR 5.22 (95% C.I. 2.86 to 9.55)	Evidence of poorer survival, and potential use as prognostic biomarker
Yu et al. (2017) [98]	*Fusobacterium*, *Anaerosporobacter*, *Parvimonas*, *Peptostreptococcus*, and *Prevotella*	Pyrosequence (Roche 454 GS FLX)	Phase I: 16 CRC with recurrence and 15 CRC without recurrence Phase II: 48 CRC without recurrence and 44 CRC with recurrence	Recurrence rate in the high-risk vs. low-risk group (73.4% vs. 30.9%, *p* < 0.001)	High amount of *F. nucleatum* could favor CRC chemoresistance and predict potential CRC recurrence
Little et al. (2019) [99]	*S. bovis*	*S. bovis*-positive blood cultures	86 patients with *S. bovis* bacteriemia	30 patients underwent colonoscopy with 3 (10%) having adenocarcinoma and 11 (37%) having adenomatous polyps. Gastroenterology consultation was significantly associated with having a colonoscopy (*p* = 0.001).	Association between *S. bovis* bacteremia and CRC risk.
Corredoira-Sánchez et al. (2012) [100]	*S. gallolyticus*	*S. gallolyticus* positive blood cultures	109 patients with *S. gallolyticus* bacteriemia and 196 controls	98 patients underwent colonoscopy: 57 had adenomas (39 advanced adenomas) and 12 had invasive carcinomas. Total colorectal neoplasia in patients with *S. gallolyticus* bacteriemia vs. controls: 70% vs. 32%; OR 5.1, 95% C.I. 3.0–8.6). For advanced adenomas: 40% vs. 16%; OR 3.5; 95% C.I. 2.0–6.1. For invasive carcinomas: 12% vs. 5%; OR 2.9, 95% C.I. 1.2–6.9.	*S. gallolyticus* infection could represent a valuable marker for detection of occult CRC
Butt et al. (2016) [101]	*S. gallolyticus*	Antibody responses to recombinant affinity-purified *S. gallolyticus* pilus proteins Gallo1569, 2039, 2178 and 2179 were analysed by multiplex serology	576 CRC and 576 controls	Antibody responses to Gallo2039 (OR 1.58, 95% C.I. 1.09–2.28), Gallo2178 (OR 1.58, 95% C.I. 1.09–2.30) and Gallo2179 (OR 1.45, 95% C.I. 1.00–2.11) were significantly associated with CRC risk. The association was stronger for positivity to two or more pilus proteins of Gallo1569, Gallo2178 and Gallo2179 (OR 1.93, 95% C.I. 1.04–3.56) and for double-positivity to Gallo2178 and Gallo2179 (OR 3.54, 95% C.I. 1.49–8.44)	Association between *S. gallolyticus* infection and CRC risk
Purcell et al. (2017) [102]	ETBF	Quantitative PCR	150 consecutive patients who underwent colonoscopy	Associations with low-grade dysplasia (*p* = 0.007), tubular adenomas (*p* = 0.027), and serrated polyps (*p* = 0.007)	Potential marker of early colorectal carcinogenesis
Xie et al. (2016) [103]	ETBF and *pks* + *E. coli*	Quantitative real time PCR	36 adenoma, 18 controls	Increase of toxin produced by ETBF in adenoma vs. controls (*p* = 0.003) and in *pks* + *E. coli* (*p* < 0.001)	Possible relationship with carcinogenesis in adenomas
Zamani et al. (2020) [104]	ETBF	Quantitative real-time PCR	68 precancerous and CRC condition, 52 controls	Positivity of *bft* gene in patients vs. controls *p* = 0.00. OR 22.22 (95% C.I. 5–98.74)	Risk factor and screening marker for developing CRC
Viljoen, et al. (2015) [105]	*Fusobacterium* spp., *Streptococcus gallolyticus*, *Enterococcus faecalis*, ETBF, Enteropathogenic *E. coli*, and afaC− or *pks* + *E. coli*	Quantitative PCR	Paired tumor and normal tissue samples from 55 CRC	*Fusobacterium* was significantly higher in CRC vs. controls (*p* < 0.001). ETBF (FDR = 0.04 and 0.002 for controls and CRC, respectively) and *Fusobacterium* spp. (FDR = 0.03 CRC) levels were significantly higher in stage III/IV CRC	Associations with clinicopathological features, mainly for *Fusobacterium* and ETBF
Ambrosi et al. (2019) [106]	*E. coli*	16S rRNA gene sequencing and PCR	Phase I: 20 adenomatous polyps, 20 polyps, 20 adjacent tissue close to polyps (5–7 cm), 10 controls Phase II: total 1500 biopsies, 600 adenomatous polyps, 600 adjacent non-adenomatous tissues, 300 controls	In polyps, prevalence of phylogroup A and B2, strong biofilm and poor protease producers (*p* < 0.05). Phylogroup B2 showed highest isolates with virulence factor score ≥10 (*p* = 0.0034).	Association of specific phenotypes of *E. coli* with adenomatous polyps
Iyadorai et al. (2020) [107]	*Pks* + *E. coli*	16S rRNA gene sequencing and PCR	Phase I: Primary colon epithelial and CRC (HCT116) cell lines Phase II: 48 CRC (48 tumor and 48 matching non-malignant tissue), 23 controls (23 proximal and 23 distal biopsies)	16.7% of CRC patients were positive for *pks* + *E. coli* vs. 4.35% of controls (*p* = 0.144). *Pks* + *E. coli* was observed in 1/26 colonoscopy biopsies from controls vs. 16/96 tissue samples from CRC (*p* = 0.01)	Initiation and development of CRC

**Abbreviations:** T-RFLP: terminal restriction fragment length polymorphism, NGS: next-generation sequencing, MGWAS: metagenome-wide association study, CA: conventional adenoma, FQ-PCR: fluorescent quantitative polymerase chain reaction, FISH: fluorescence in situ hybridization, C.I.: confidence interval, qRT-PCR: real-time quantitative reverse transcription polymerase chain reaction, HP: proximal hyperplastic polyp, SSA: sessile serrated adenoma, TA: traditional adenoma, HR: hazard ratio, OR: odds ratio, ETBF: enterotoxigenic *Bacteroides fragilis*, afaC: afimbrial adhesin, *pks*: polyketide synthase, FDR: false discovery rate.
